# Sensitivity of spiral ganglion neurons to damage caused by mobile phone electromagnetic radiation will increase in lipopolysaccharide-induced inflammation *in vitro* model

**DOI:** 10.1186/s12974-015-0300-1

**Published:** 2015-05-29

**Authors:** Wen-Qi Zuo, Yu-Juan Hu, Yang Yang, Xue-Yan Zhao, Yuan-Yuan Zhang, Wen Kong, Wei-Jia Kong

**Affiliations:** Department of Otolaryngology, Union Hospital, Tongji Medical College, Huazhong University of Science and Technology, Wuhan, 430022 People’s Republic of China; Institute of Otorhinolaryngology, Union Hospital, Tongji Medical College, Huazhong University of Science and Technology, Wuhan, 430022 People’s Republic of China; Department of Endocrinology, Union Hospital, Tongji Medical College, Huazhong University of Science and Technology, Wuhan, 430022 People’s Republic of China

**Keywords:** Lipopolysaccharide, Spiral ganglion neurons, Sensitivity, Damage, Radiofrequency electromagnetic radiation

## Abstract

**Background:**

With the increasing popularity of mobile phones, the potential hazards of radiofrequency electromagnetic radiation (RF-EMR) on the auditory system remain unclear. Apart from RF-EMR, humans are also exposed to various physical and chemical factors. We established a lipopolysaccharide (LPS)-induced inflammation *in vitro* model to investigate whether the possible sensitivity of spiral ganglion neurons to damage caused by mobile phone electromagnetic radiation (at specific absorption rates: 2, 4 W/kg) will increase.

**Methods:**

Spiral ganglion neurons (SGN) were obtained from neonatal (1- to 3-day-old) Sprague Dawley® (SD) rats. After the SGN were treated with different concentrations (0, 20, 40, 50, 100, 200, and 400 μg/ml) of LPS, the Cell Counting Kit-8 (CCK-8) and alkaline comet assay were used to quantify cellular activity and DNA damage, respectively. The SGN were treated with the moderate LPS concentrations before RF-EMR exposure. After 24 h intermittent exposure at an absorption rate of 2 and 4 W/kg, DNA damage was examined by alkaline comet assay, ultrastructure changes were detected by transmission electron microscopy, and expression of the autophagy markers LC3-II and Beclin1 were examined by immunofluorescence and confocal laser scanning microscopy. Reactive oxygen species (ROS) production was quantified by the dichlorofluorescin-diacetate assay.

**Results:**

LPS (100 μg/ml) induced DNA damage and suppressed cellular activity (*P* < 0.05). LPS (40 μg/ml) did not exhibit cellular activity changes or DNA damage (*P* > 0.05); therefore, 40 μg/ml was used to pretreat the concentration before exposure to RF-EMR. RF-EMR could not directly induce DNA damage. However, the 4 W/kg combined with LPS (40 μg/ml) group showed mitochondria vacuoles, karyopyknosis, presence of lysosomes and autophagosome, and increasing expression of LC3-II and Beclin1. The ROS values significantly increased in the 4 W/kg exposure, 4 W/kg combined with LPS (40 μg/ml) exposure, and H_2_O_2_ groups (*P* < 0.05, 0.01).

**Conclusions:**

Short-term exposure to radiofrequency electromagnetic radiation could not directly induce DNA damage in normal spiral ganglion neurons, but it could cause the changes of cellular ultrastructure at special SAR 4.0 W/kg when cells are in fragile or micro-damaged condition. It seems that the sensitivity of SGN to damage caused by mobile phone electromagnetic radiation will increase in a lipopolysaccharide-induced inflammation *in vitro* model.

## Background

In recent decades, the increasing mobile phones are widely used in our life, which have raised public concerns about the potential hazards to human health. According to system needs, mobile phone frequencies range from 900 to 2,200 MHz, which belong to the radiofrequency (RF) spectrum [1]. Bio-effects of radiofrequency electromagnetic radiation (RF-EMR) have been reported (for example, impaired male fertility [2], decrease in the neuronal cell numbers which affects brain activity [3], increased risk of glioma and acoustic neuroma [4], induced repairable DNA damage in human Liver Engrafting Cells (hLEC™) and Chinese hamster lung cells [5]), and many other negative findings [6,7] are also reported in some studies.

More than other areas of the body, the ears are frequently exposed and in very close proximity to the mobile phone; the potential bio-effect of RF-EMR on ears maybe more serious than on other organs. Actually, the bio-effect on ears remains controversial, because the cochlear is deep in the temporal bone, electromagnetic effects may be weakened; some studies show negative results in *in vivo* model [8]. Furthermore, after exposure to 1,763-MHz RF radiation, there was no significant damage to or changes in the cell cycle, DNA, gene expression, and stress response in HEI-OC1 mouse auditory hair cell [9]. These findings demonstrated that the bio-effects may be associated with the differential sensitivities of cell lines and tissues to RF-EMR, because the photons of RF-EMR have insufficient quantum energy to cause ionizations in atoms, so RF-EMR is not considered to induce direct DNA damage [10]. However, apart from RF-EMR, humans are also exposed daily to various environmental factors (for example, chemicals, UV radiation, ultrafine particles) that cause unfavorable effects such as bio-effects of RF-EMR which might be enhanced by environmental factors.

Otitis media (OM) is a common otolaryngological disease and difficult to cure. Lipopolysaccharide (LPS), a bacterial toxin, was frequently detected in the effusion from otitis media of patients [11]. Inflammation may often damage the structure (for example, spiral ganglion neurons (SGN), inner and outer hair cells, and marginal cells) of the inner ear through the round window membrane and cause sensorineural hearing loss [12]. In the inner ear, the spiral ganglion neurons serve the important function of conveying electric signals into the brain, and normal functioning of spiral ganglion is presumed in cochlear implant therapy for hearing loss. Once the spiral ganglion neurons are damaged, hearing impairment can occur. In this sense, it would be very important to know whether any biological alterations (for example, DNA damage, ultrastructure changes, protein expression) in spiral ganglion neurons can occur by mobile phone radiofrequency exposure.

The irradiation-induced generation of reactive oxygen species (ROS) is considered to be one of the primary mechanisms that participate in the bio-effects that are mediated by RF-EMR exposure [13]. Some previous studies have demonstrated that RF-EMR increased the formation of ROS both *in vivo* and *in vitro*, which have been shown to induce DNA oxidative damage [5,14,15]. Many studies have indicated that ROS production is an important intracellular inducer of autophagy [16,17]. LC3-II and Beclin1 are considered to be biomarkers of autophagy [18].

In the present study, we established a LPS-induced inflammation *in vitro* model to investigate whether the possible sensitivity of spiral ganglion neurons to damage caused by mobile phone electromagnetic radiation will increase. We checked several parameters, such as DNA damage, changes in ultrastructure, expression of LC3-II and Beclin1, and ROS production.

## Materials and methods

### Animal

The neonatal (1- to 3-day-old) Sprague Dawley (SD) rats were purchased from the animal center of the ChongQing Medical University. The animal center is officially qualified to raise and breed research animals. Animals were fed according to the standard protocols approved by the Statute of Laboratory Animal Management Administration of China and the procedure was approved by the Ethics Committee of the ChongQing Medical University.

### Primary culture and identification of SGN

Ten neonatal SD rats were treated with 75% ethanol after receiving ethyl-ether-inhaled anesthesia. The bilateral temporal bones were removed, and the spiral ganglion tissue was dissected form the stria vascularis and basilar membrane; then, the tissue was digested with 0.25% trypsin for 30 min at 37°C. After the samples were centrifuged for 8 min at 800 rpm, the cells were resuspended and placed in 35-mm cell culture plates coated with poly-L-lysine. Cells were maintained in a growth medium with DMEM/F12 (Hyclone, Logan, UT, USA), supplemented with 20% fetal bovine serum (GIBCO, Carlsbad, CA, USA), 10% neurotrophic factors B27, and 1% penicillin-streptomycin and placed in an incubator at 37°C in 5% CO2 and 95% air. We used 5 μmol/ml cytarabine to purify the SGN for 72 h. Neuronal class III anti-β-tubulin antibody (TUJ1, 1:200)(Santa Cruz, Dallas, TX, USA) was used to identify neurons. Immunofluorescence procedures were performed according to the following steps. The SGN were digested by 0.25% trypsin and mounted onto glass microscope slides, which were treated with poly-L-lysine and sterilized ultraviolet rays for 15 min. The SGN were washed with phosphate-buffered saline (PBS, pH 7.4) once. Next, the SGN were fixed in precooled 4% paraformaldehyde for 15 min at room temperature and washed once in PBS for 5 min, permeabilized in 0.3% Triton™ X-100 (Sigma, St. Louis, MO, USA) for 15 min, and then washed in PBS once. Subsequently, the specimens were blocked in a solution of 5% bovine serum albumin (BSA) for 10 min. The specimens were incubated overnight at 4°C in a solution (1:200) containing anti-β-III tubulin (TUJ1), a mouse monoclonal antibody (Santa Cruz, Dallas, TX, USA). After being washed three times in PBS, the specimens were incubated with anti-mouse IgG (diluted 1:200 with 1% BSA) for 30 min at 37°C. After processing for immunofluorescence, the slides were mounted with glycerinum and observed using a confocal laser scanning microscope (CLSM) (Nikon, Tokyo, Japan).

### LPS-induced cytotoxicity and cellular activity as detected by CCK-8

SGN were digested and seeded into a 96-well growth-medium plate, and after adhering to the plates after 12 h, the SGN were treated with different concentrations of LPS (0, 20, 40, 50, 100, 200, and 400 μg/ml) and incubated for 48 h. Three replicative wells were detected for each concentration. After 48 h, the SGN were washed twice with PBS and given fresh complete growth medium. Then, 20 μl of the Cell Counting Kit-8 (CCK-8; Dojindo, Kumamoto, Japan) test solution was added to each well, and the cells were incubated at 37°C for 1.5 h. The 0 μg/ml group was defined as the control group. The absorbance value (OD value) at the 450-nm wavelength was measured using a microplate reader. The cell activity rate (%) = (OD experimental − OD blank)/(OD control − OD blank) × 100.

### LPS-induced DNA damage as detected by alkaline comet assay

The alkaline comet assay (pH > 13) is widely used to quantify DNA damage, such as single-strand breaks (SSB), double-strand breaks (DSB), and alkali labile sites (ALS), especially in SSB [19]. It has proven to be a sensitive method of measuring the induction and repair of DNA damage in individual cells [5,20-22]. After cells were exposed to LPS (0, 20, 40, 50, 100, 200, 400 μg/ml) and H_2_O_2_, they were washed with PBS, trypsinized, and resuspended in PBS for the comet assay. The cell suspension (10 μl: 1,000 cells) was mixed with 60 μl LMPA (0.65% low-melting-point agarose) and immediately pipetted onto Trevigen CometSlides™ (Trevigen, Gaithersburg, MD, USA). After the slides were precooled at 4°C for 30 min in the dark and agarose had solidified, they were immersed in the lysing solution (2.5 M NaCl, 100 mM Na_2_EDTA, 10 mM Tris, 1% Triton X-100, 10% DMSO, pH 10) for 2 h at 4°C in the dark. After the slides were allowed to unwind for 30 min in an alkaline buffer (1 mM Na_2_EDTA, 300 mM NaOH, pH 13) at room temperature, they were subjected to electrophoresis at 25 V/300 mA for 40 min. The slides were washed twice in dH2O for 5 min and dehydrated in 95% and 75% ethanol for 5 min each. Subsequently, the slides were air-dried in the dark at 4°C. Then, these slides were dyed with attenuated SYBR® Green I (Invitrogen, Waltham, MA, USA) and examined at 200× magnification with a Leica fluorescence microscope (Leica Microsystems, Wetzlar, Germany). The tail DNA (%), tail moment (arbitrary units), and tail length (μm) were used to express the level of DNA damage [23]. Images of 200 randomly selected cells were analyzed from each slide. All of the comet parameters were assessed by a comet assay image analysis system (Comet Assay Software Project, CASP Lab, Poland).

### SGN were treated with LPS before exposure to RF-EMR

Using the procedures described above, we treated the SGN with LPS (40 μg/ml) for 24 h before exposure to RF-EMR. The cellular activity and DNA exhibited no obvious changes after treatment with LPS (40 μg/ml). Therefore, 40 μg/ml was used to pretreat the concentration before exposure to RF-EMR.

### RF-EMR exposure system

The RF-EMR exposure system was built by the Foundation for Information Technologies in Society (IT’IS Foundation, Zurich, Switzerland), as described in detail in previous studies [24-26]. This exposure system consists of four primary parts: two rectangular waveguides, an RF generator, a narrow-band amplifier, and an arbitrary function generator. One waveguide is used for the radiation-exposure groups, whereas the other waveguide is used for the sham-exposure group. Both of these waveguides were placed inside a CO_2_ incubator. The exposure system was connected to a computer that controlled the specific absorption rate (SAR) value during the exposure process and maintained the temperature of the incubator at 37°C, 5% CO_2_/95%. The SAR variability of this exposure system was controlled below 6%, with a temperature rise of 0.03°C/(W/kg) of the average SAR value following exposure of monolayer cells. This system operated under the stable frequency of 1,800 MHz. Six 35-mm Petri dishes can be placed in the H-field maxima and exposed to polarized E field (an electric field that is perpendicular to the H field) in the same manner. The temperature change is uniformly distributed in monolayer cells, and the temperature difference between the RF-exposed and sham-exposed chambers did not exceed 0.1°C [27]. All of the exposure parameters and monitor data (for example, temperature, SAR value) were encoded and recorded in a data file, which was transmitted to the IT’IS Foundation by e-mail and decoded by the foundation for the data analysis.

### Cell-exposure procedure and groups

After SGN were treated with 40 μg/ml LPS for 24 h, the culture medium was renewed, and cells were subjected to a 24-h exposure to 1,800 MHz GSM ‘talk mode’ signals with an intermittent cycle of 5 min on and 10 min off. To explore the SAR-related effects of RF radiation exposure, dishes were randomly divided into the following groups: (1) RF radiation control, (2) 2 W/kg (either sham-exposure or exposure), (3) 4 W/kg (either sham-exposure or exposure), (4) 2 W/kg combined with LPS (40 μg/ml) (either sham-exposure or exposure), and (5) 4 W/kg combined with LPS (40 μg/ml) (either sham-exposure or exposure).

### RF-EMR alone or combined LPS induce DNA damage detected by alkaline comet assay

Following exposure to RF-EMR and LPS (40 μg/ml), we applied alkaline comet assay to all groups to detect DNA damage, using the steps as described in the above comet assay procedure.

### Cellular ultrastructure changes detected by transmission electron microscopy (TEM)

After exposure to RF-EMR and LPS (40 μg/ml), cells were detached by 0.25% trypsin digestion, and samples were centrifuged for 10 min at 1,200 rpm. Using a straw to draw supernatant after centrifugation, we mixed the precooled 4% glutaraldehyde stationary liquid slowly, after 1- to 1.5-h incubation at 4°C; the samples were fixed, dehydrated, embedded, and sectioned. Ultrathin sections were observed under H-7500 transmission electron microscope (TEM).

### Expression of autophagy indicator in SGN as detected by immunofluorescence

We examined the expression of autophagy indicators in the above cell-exposure groups. Using an immunofluorescence method and CLSM (Nikon, Tokyo, Japan), we detected the distribution of LC3-II and Beclin1 in SGN. The same steps as described in the above immunofluorescence procedure were used with primary antibody LC3-II and Beclin1 (1:100, abcam, Cambridge, MA, USA). After the samples were incubated with the green and red secondary antibody (1:200) for 60 min, the slides were treated with 4′,6-diamidino-2-phenylindole (DAPI) for 5 min. Then, the samples were mounted and observed by CLSM.

### Assay of intracellular ROS

Dichloro-dihydro-fluorescein diacetate (DCFH-DA) (Beyotime Company, Shanghai, China) was applied to detected intracellular oxidant production. Eleven experimental or control groups were assigned to assay ROS: (1) control group, (2) LPS (40 μg/ml) group, (3) 2-W/kg (either sham-exposure or exposure), (4) 4 W/kg (either sham-exposure or exposure), (5) 2-W/kg combined with LPS (40 μg/ml) (either sham-exposure or exposure), (6) 4 W/kg combined with LPS (40 μg/ml) (either sham-exposure or exposure), and (7) H_2_O_2_ groups. Briefly, after exposed to LPS (40 μg/ml) and RF-EMR, the SGN were placed in the fluorescent probe then incubated with DCFH-DA (1:1,000) 1 ml at 37°C for 30 min; the control group was incubated with DMEM/F12 medium. Then, the SGN were washed three times with the medium. The amount of emitted fluorescence was correlated with the quality of ROS in the SGN. The fluorescence value was acquired with a fluorescence photometer.

### Statistical analysis

All the results were expressed as means ± standard error of the mean (SEM). SPSS 17.0 was used to analyze the results. One-way ANOVA and two-tailed independent-sample *t*-test were used to evaluate the differences in activity among the different concentrations of LPS-treated groups and the control group at the same time, respectively. Based on the findings in previous studies, median values of DNA damage were selected to represent the amount of DNA damage in our findings. Therefore, comparisons of the averages of the median DNA damages for the different concentrations of LPS and radiation-exposed and sham-exposed control groups were compared using the two-tailed Student *t*-test, as described in previous studies [21,28]. Differences exhibiting *P* < 0.05 were considered to be statistically significant.

## Results

### Detection of SGN *in vitro*

**Figure 1:**
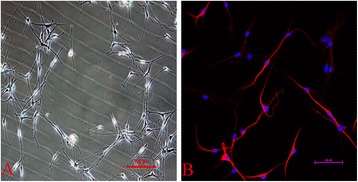
Morphology and identification of SGN. **(A)** The morphology of SGN. This picture shows the SGN, which are comprised of elliptical nuclei and bipolar axons (shown at 100× magnification). **(B)** The test for β-III-tubulin (TUJ1) by immunofluorescence in the SGN. TUJ1 is mainly expressed in cytomembrane and axons. The nuclei were stained with DAPI (1:10,000), as indicated by blue fluorescence (400× magnification).

As shown in Figure 1A, SGN can be distinguished from fibroblasts under an inverted phase contrast microscope based on their typical bipolar neurons. The SGN cell bodies are oval and smooth, and the axons extend out from the cell body as slender projections. Figure 1B shows that an anti-β-III tubulin (TUJ1) fluorescent biomarker signal could be detected in the cultured cells.

### LPS-induced cytotoxicity and cellular activity

**Figure 2:**
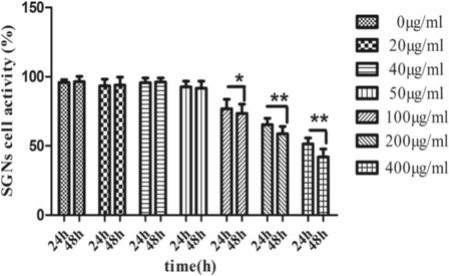
LPS-induced cytotoxicity and cellular activity was measured by CCK-8. The 0 μg/ml group was defined as the control group. Compared with the control group, there were no significant statistical differences between the mean OD values of the 20, 40, and 50 μg/ml groups after 24 and 48 h. This might indicate that the SGN grew well under these three concentration levels of LPS. However, the mean OD values of the other groups (100, 200, 400 μg/ml) were less than that of the control group. The *P* values for both the 24- and 48-h exposures for the 100, 200, and 400 μg/ml groups were less than 0.05. The result indicated that the activity of the SGN was suppressed when the LPS concentration was over 50 μg/ml. The data are presented as the means ± SEM. *P < 0.05 compared to the control group; **P < 0.01 compared to the control group.

LPS-induced cytotoxicity, expressed as a change in cellular activity, is shown in Figure 2. The cellular activity of the control group was identified as 100%, indicating no cytotoxicity. With an increase in concentration of LPS, the cell activity gradually declines. At 100 μg/ml concentration, indicated as critical concentration, the SGN activity was significantly suppressed. According to the results of the CCK-8, we chose 40 μg/ml as the concentration of the pretreatment before RF-EMR. This might indicate that the SGN grew well under this concentration level of LPS.

### Alkaline comet assay

**Figure 3:**
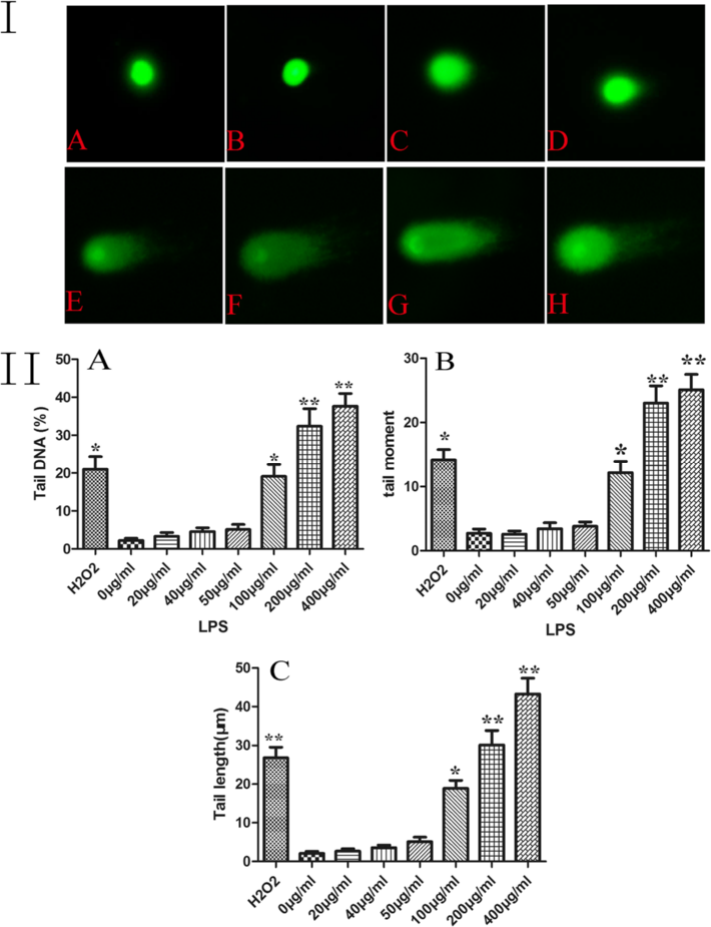
LPS induce DNA damage (I), and comet assay’s parameters were analyzed by CASP software (II). **(I)** A. control group; B. 20 μg/ml group; C. 40 μg/ml group; D. 50 μg/ml group; E. 100 μg/ml group; F. 200 μg/ml group; G. 400 μg/ml group; and H. H_2_O_2_-positive group. The DNA parameters were analyzed by CASP software, and values were shown in part II. **(II)** SGN were treated with different concentrations of LPS for 48 h or H_2_O_2_ (30 μM) for 15 min. The levels of DNA damage were expressed as the tail DNA (%) (A), tail moment (B), and tail length (μm) (C). The H_2_O_2_ group was defined as the positive group. *P < 0.05, **P < 0.01 compared to the control group. Two hundred nuclei were analyzed for each group of experiment. LPS, lipopolysaccharide.

**Figure 4:**
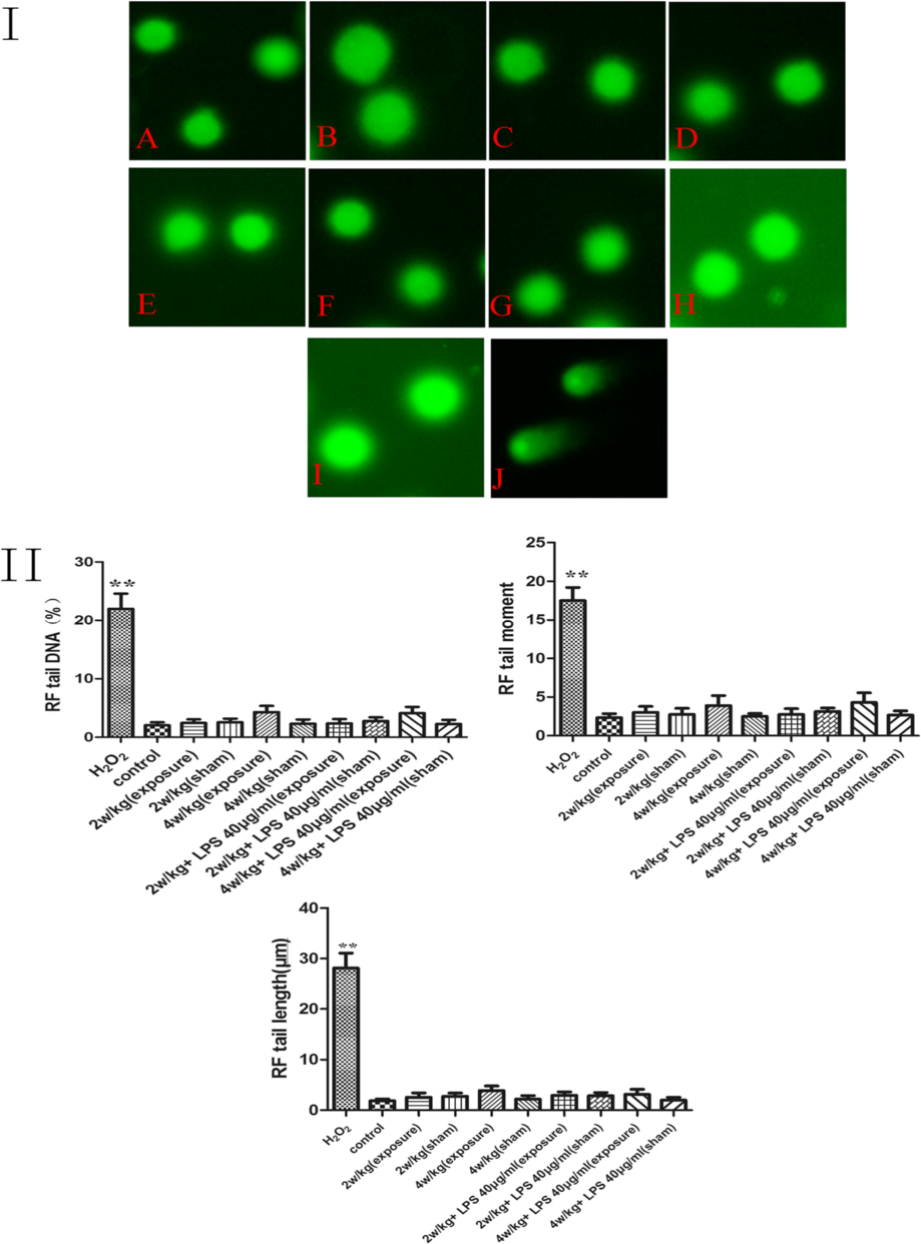
RF-EMR alone or combined with LPS (40 μg/ml) did not induce DNA damage (I), and comet assay’s parameters were analyzed by CASP software (II). **(I)** A. RF radiation control group; B. 2 W/kg sham exposure group; C. 2 W/kg exposure group; D. 4 W/kg sham exposure group; E. 4 W/kg exposure group; F. 2 W/kg combined with LPS (40 μg/ml) sham exposure group; G. 2 W/kg combined with LPS (40 μg/ml) exposure group; H. 4 W/kg combined with LPS (40 μg/ml) sham exposure group; I. 4 W/kg combined with LPS (40 μg/ml) exposure group; and J. H_2_O_2_ group. The parameters of DNA were analyzed by CASP software, and values are shown in part II. **(II)** The H_2_O_2_ group was defined as the positive group. **P < 0.01 compared to the control group. There were no significant differences between all of groups’ DNA parameters (*P* > 0.05), indicating that RF radiation could not directly induce DNA damage. LPS, lipopolysaccharide; RF, radiofrequency.

The body of normal cells exhibit intact, undamaged DNA that remains in the region of the nuclear matrix. The damaged cell, on the other hand, assumes a comet-like appearance, including a ‘tail’ shape. These results showed that exposure to H_2_O_2_ and LPS (at 100, 200, 400 μg/ml) significantly increased all the comet appearance parameters, which indicated that the dose of LPS and H_2_O_2_ could effectively result in DNA strand breaks (Figure 3). However, after exposure to radiation at 2 and 4 W/kg with and without combination with LPS (40 μg/ml), no significant difference in the comet parameters was found among any of the groups (Figure 4). It seems, thus, that RF-EMR could not directly lead to DNA strand breakage.

### RF-EMR-induced cell ultrastructure damages and autophagy

**Figure 5:**
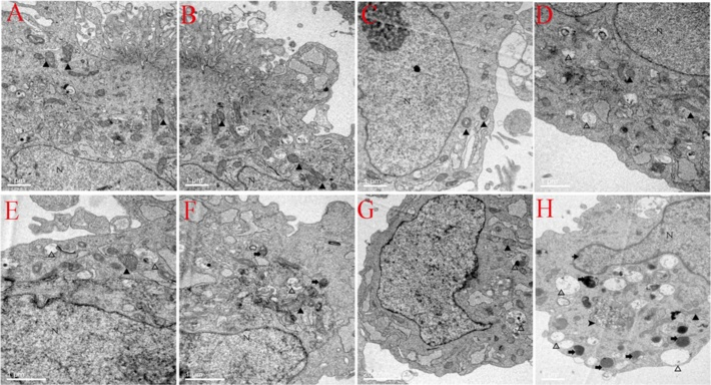
RF-EMR-induced cellular ultrastructure changes. **(A)** 2 W/kg sham-exposure group; **(B)**. 2 W/kg exposure group; **(C)** 4 W/kg sham-exposure group; **(D)** 4 W/kg exposure group; **(E)** 2 W/kg combined with LPS (40 μg/ml) sham-exposure group; **(F)** 2 W/kg combined with LPS (40 μg/ml) exposure group; **(G)** 4 W/kg combined with LPS (40 μg/ml) sham-exposure group; and **(H)** 4 W/kg combined with LPS (40 μg/ml) exposure group. Pictures A to G of ultrastructure show no obvious changes. However, in H picture, SGN’s cellular ultrastructure shows mitochondria vacuoles (show *empty triangle*), karyopyknosis (*thin arrow*), lysosome (*thick arrow*), and autophagosome (*arrow head*), *black triangle*: normal mitochondria, N: nucleus.

**Figure 6:**
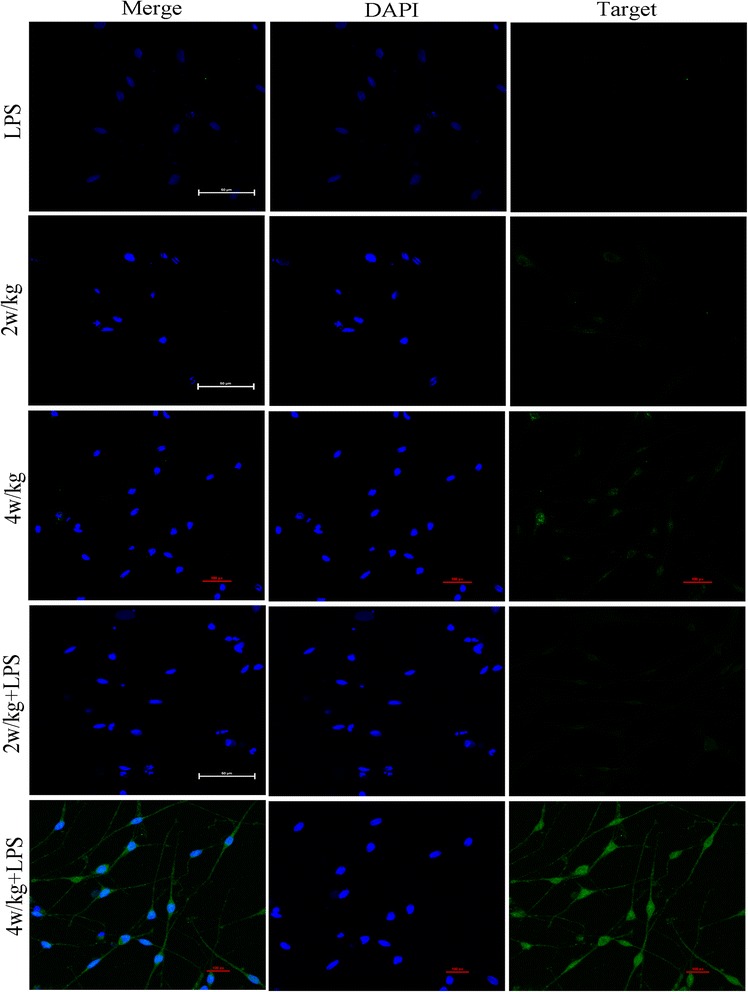
RF-EMR-induced cellular autophagy. The expression of LC3-II show no statistically significant changes in LPS (40 μg/ml) group, 2 W/kg sham and exposure group, 4 W/kg sham and exposure group, 2 W/kg combined with LPS (40 μg/ml) sham-exposure and exposure group, and 4 W/kg combined with LPS (40 μg/ml) sham-exposure group. However, after 4 W/kg combined with LPS (40 μg/ml) exposure RF-EMR, the expression of LC3-II is significantly higher than other groups. DAPI, 4′,6-diamidino-2-phenylindole; LPS, lipopolysaccharide.

**Figure 7:**
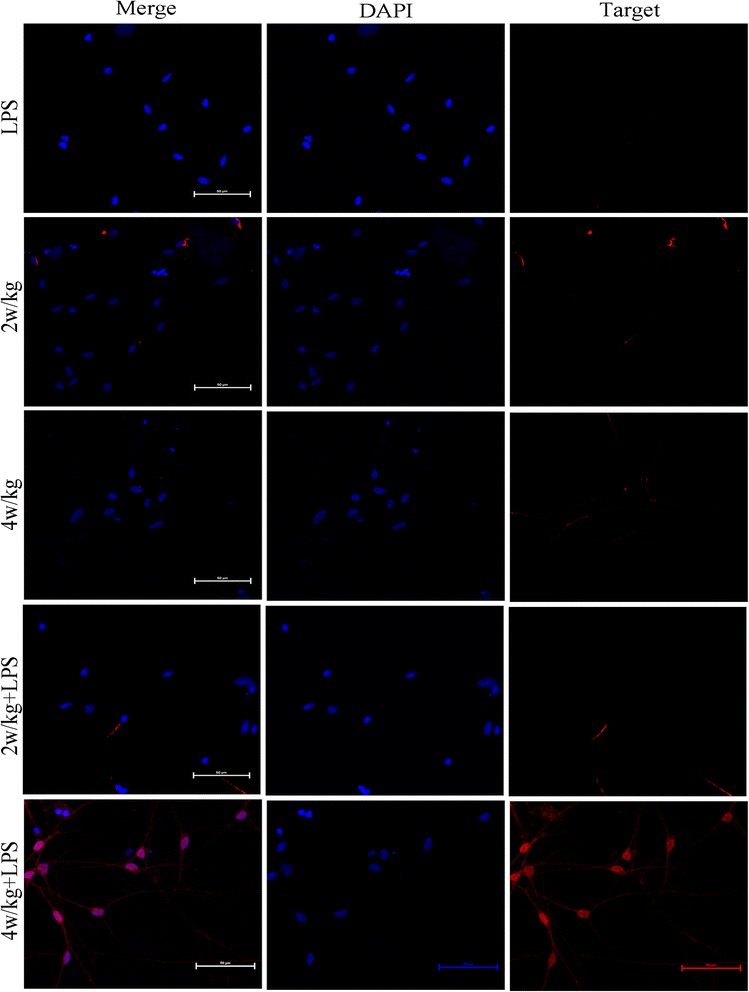
RF-EMR-induced cellular autophagy. The expression of Beclin1 show no statistically significant changes in LPS (40 μg/ml) group, 2 W/kg sham and exposure group, 4 W/kg sham and exposure group, 2 W/kg combined with LPS (40 μg/ml) sham-exposure and exposure group, and 4 W/kg combined with LPS (40 μg/ml) sham-exposure group. However, after 4 W/kg combined with LPS (40 μg/ml) exposure RF-EMR, the expression of Beclin1 is significantly higher than other groups. DAPI, 4′,6-diamidino-2-phenylindole; LPS, lipopolysaccharide.

After SGN exposure to RF-EMR and LPS (40 μg/ml), we detected cellular ultrastructural changes by TEM, such as mitochondrial vacuoles, karyopyknosis, and presence of lysosomes and autophagosome in 4 W/kg combined with LPS (40 μg/ml) in group (Figure 5). In order to further confirm whether RF-EMR could induce autophagy in the SGN, we observed the expression of LC3-II (Figure 6) and Beclin1 (Figure 7) (autophagy indicators) by immunofluorescence. The expression of LC3-II and Beclin1 increased and was significantly higher in the group exposed to radiation at 4 W/kg combined with LPS (40 μg/ml) group.

### ROS are involved in RF radiation-induced ultrastructure damages and autophagy

**Figure 8:**
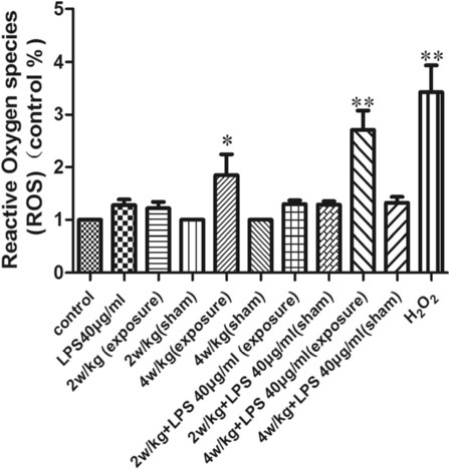
ROS production in primary SGN exposed to RF-EMR and LPS. After exposure to RF-EMR and LPS, SGN were incubated with DCFH-DA at 37°C for 30 min. Cellular fluorescence intensity was expressed as the multiple of the level in control groups. **P < 0.01 compared to the control group, *P < 0.05 compared to the control group. Values are means ± SE, *n* = 12. LPS, lipopolysaccharide.

Fluorescence images were acquired with a Leica fluorescence microscope, using 485 nm for excitation and 530 nm for emission, and images were analyzed for pixel intensity by NIH Image. Cellular fluorescence intensity was expressed as the multiple of the level in control groups. As shown in Figure 8, after SGN exposed either to H_2_O_2_ or to 4 W/kg with and without combination with LPS (40 μg/ml), their ROS levels were significantly increased in a dose-dependent manner.

## Discussion

RF-EMR could not directly induce DNA damage of spiral ganglion neurons in all of the groups. The result is consistent with the majority of previous studies. However, we found that cellular ultrastructure changes autophagy and ROS overproduction in 4 W/kg combined with LPS (40 μg/ml) group.

SGN receive electrical signal input from cochlear hair cells and project from the cochlea to the cochlear nucleus; then, the electrical signals were transmitted to the auditory cortex [29]. So, the SGN were referred to as the first level of neurons of the auditory system. The dysfunction of SGN often leads to sensorineural hearing loss and causes implantable hearing device failure. Radiofrequency exposure induces ROS generation, which leads changes in cell membrane function, cellular signal communication and metabolism, and causes nerve cell death [30]. However, relevant data are lacking regarding the consequence of RF-EMR exposure on cochlear spiral ganglion tissue or neurons.

In our study, we found that RF-EMR could not induce DNA damage and cellular ultrastructure changes in normal SGN. On the one hand, the energy of RF-EMR is ostensibly too weak to break DNA directly [5]. On the other hand, the normal SGN is tough, so it is insusceptible to the damage caused by electromagnetic radiation. However, in the lipopolysaccharide-induced inflammation *in vitro* model, we found that cellular ultrastructure changes, such as mitochondrial vacuoles, karyopyknosis, and presence of lysosomes and autophagosome, are considered to be an indicator of cell damage [31]. We consider that the SGN maybe in a fragile state after pretreatment with low-concentration pretreatment of LPS (40 μg/ml), and the sensitivity of SGN to damage caused by 4 W/kg radiation is increased in this condition. LPS induced synthesis of reactive nitric oxide (NO), prostaglandin E2 (PGE_2_), and inflammatory cytokines, which interacts with biological molecules and damages cell membranes resulting in cell death [32]. LPS was frequently detected in the effusion from otitis media of patients [11]. With increases in concentration of LPS exposure, we found that LPS (100 μg/ml) can induce cellular DNA damage and decrease of the cell vitality. However, cellular DNA and vitality did not change in LPS (40 μg/ml), so 40 μg/ml concentration was used as a pretreatment before exposure.

Some researchers have successfully set up the animal model of otitis media [33], but the inner ear is located deep in the temporal bone; the progress of inflammation and related indicators are difficult to observe; therefore, in this study, we choice an *in vitro* model to explore the biological effect of electromagnetic radiation, but we will try to investigate the possible cooperative effects of RF-EMR and LPS on any biological alterations in an *in vivo* model in further research.

Lysosomes and autophagosome were detected in cellular ultrastructure, so we detected the expression of the autophagy biomarkers LC3-II and Beclin1 and found them to be significantly increased in 4 W/kg combined with LPS group. Autophagy is a protective cellular process, especially following acute damage, but excessive autophagy can also lead to cell death [34]. Liu et al. [27] found that autophagy may play an important role in preventing mouse spermatocyte-derived cells from apoptotic cell death after exposure to 1,800-MHz GSM exposure (4 W/kg), but following this experiment, the role of autophagy still needs further research.

Activating the ROS system is thought to be the main mechanism of electromagnetic radiation biological effects, especially those resulting from acute exposure to the RF-EMR fields of mobile phones [35]. As is well known, the overproduction of ROS could cause severe damage to cellular macromolecules, and ROS production is an important intracellular inducer of autophagy. In addition, experiments have demonstrated that a SAR value for a given RF-EMR frequency, and the biological effects, are intensity related. The intracellular levels of ROS following radiation at 4 W/kg were significantly higher than those at 2 W/kg, and they more easily induce cell damage. We speculate that surplus ROS may disturb the balance between the oxidation and reduction system and lead to ultrastructure damage and autophagy.

The widely used Global System for Mobile Communications (GSM) operates at the 1,800-MHz RF band to provide cellular network phone service. According to the criteria of the Institute of Electrical and Electronics Engineers standard, the SAR limit value of mobile phones is 2 W/kg in most European countries, but in the United States, it is 1.6 W/kg [36]. In addition, following the guideline of the International Commission on Non-Ionizing Radiation Protection (ICNIRP), the whole-body average and localized (head and trunk) SAR of occupational and general public exposure are 0.4, 10, 0.08, and 2 W/kg, respectively [37]. In addition, the accepted SAR threshold for non-thermal and thermal effects is 4 W/kg, with a temperature rise of approximately 0.08°C. In our experiment, using the above international standards as a reference, we applied the SXc-1800 MHz RF-EMR exposure system that is characteristic of typical GSM ‘talk mode’ signals to investigate the bio-effects of RF-EMR at special SAR 2 and 4 W/kg. This GSM ‘talk mode’ is considered to closely resemble real-life exposure conditions (at an intermittent exposure with 5 min on and 10 min off for 24 h), and intermittent exposure may cause the greatest biological effect [38]. Intermittent, rather than continuous, exposure may more closely simulate real-world exposure to mobile phone radiation, and this exposure pattern has been examined in previous studies [39].

## Conclusions

Short-term exposure to RF-EMR could not directly induce DNA damage in normal spiral ganglion neurons, but RF-EMR could cause the changes of cell ultrastructure at special SAR 4.0 W/kg when cells are in fragile or micro-damage condition. It seems that the sensitivity of SGN to damage caused by mobile phone electromagnetic radiation will increase in a lipopolysaccharide-induced inflammation *in vitro* model. However, whether these alterations cause cochlear function disorders still need further research. Activating the ROS system is thought to be the main mechanism of electromagnetic radiation biological effects.

## Abbreviations

ANOVA: analysis of variance
BSA: bovine serum albumin
CLSM: confocal laser scanning microscopy
DAPI: 4′,6-diamidino-2-phenylindole
GSM: Global System for Mobile Communications
LPS: lipopolysaccharide
PBS: phosphate-buffered saline
PGE_2_: prostaglandin E2
RF: radiofrequency
RF-EMR: radiofrequency electromagnetic radiation
ROS: reactive oxygen species
SAR: specific absorption rate
SD: Sprague Dawley
TEM: transmission electron microscopy
